# How useful is contrast-enhanced MRI in the long-term surveillance of glioma? A multicentre retrospective longitudinal cohort study

**DOI:** 10.1007/s00330-024-11333-y

**Published:** 2025-02-28

**Authors:** Marcus Cakmak, Sepehr Mohammadian, Vera C. W. Keil, Joost W. Schouten, Philip C. de Witt Hamer, Thijs van der Vaart, Rutger K. Balvers, Ivar J. H. G. Wamelink, Frederik Barkhof, Martin van den Bent, Mark Vries, Marion Smits

**Affiliations:** 1 Department of Radiology and Nuclear Medicine, Amsterdam University Medical Centre, Amsterdam, The Netherlands; 2 Department of Radiology and Nuclear Medicine, University Medical Centre Rotterdam, Rotterdam, The Netherlands; 3 Department of Neurosurgery, University Medical Centre Rotterdam, Rotterdam, The Netherlands; 4 Brain Tumour Centre, Erasmus MC Cancer Institute, Rotterdam, The Netherlands; 5 Brain Tumour Centre, Cancer Centre Amsterdam, Amsterdam University Medical Centre, Amsterdam, The Netherlands; 6 Department of Neurosurgery, Amsterdam University Medical Centre, Amsterdam, The Netherlands; 7 Department of Neurology, University Medical Centre Rotterdam, Rotterdam, The Netherlands; 8 Department of Medical Physics and Biomedical Engineering, University College London, London, United Kingdom; 9 Department of Radiology and Nuclear Medicine, Spaarne Gasthuis, Hoofddorp, The Netherlands; 10 Medical Delta, Delft, The Netherlands

**Keywords:** Glioma, Contrast media, Magnetic resonance imaging, Disease progression

## Abstract

**Objective:**

To examine whether MRI with routine gadolinium-based contrast agent (GBCA) administration in the long-term surveillance of adult-type diffuse glioma identifies tumour progression earlier than T2-weighted (T2w) and/or T2w fluid-attenuated inversion recovery (FLAIR) MRI only.

**Materials and methods:**

In this longitudinal retrospective multicentre cohort study patients with histopathologically confirmed adult-type diffuse glioma and at least two years survival after diagnosis in 2009–2010 were included. Progression was determined by the treating physician or during the multidisciplinary team meeting and defined as the moment a change in treatment or follow-up was required. The primary outcome was the proportion of patients that showed an increase of abnormalities on both contrast-enhanced T1-weighted (CET1w) and T2w/T2w-FLAIR at the time of progression. Chi-square testing was performed to analyse the relationship between the detection of progression on both scan sequences, with calculating the *Phi* coefficient to determine the degree of association.

**Results:**

One hundred eight consecutive patients were included (58 male; 53 grade 2, 21 grade 3, 34 grade 4). Progression was present in 82 patients and was determined on both CET1w and T2w/T2w-FLAIR images in 59 patients (72.0%). In 20 patients (24.4%), progression was determined based solely on T2w/T2w-FLAIR abnormalities. Only three patients showed progression exclusively on CET1w (3.7%). There was a strong positive significant relationship between the detection of progression on both scan types (*p* < 0.001; *Phi* = 0.467).

**Conclusion:**

An increase in CET1w abnormalities was generally accompanied by an increase in T2w/T2w-FLAIR abnormalities, raising the question of whether routine administration of GBCA is always necessary for long-term survivors of glioma.

**Key Points:**

***Question*** Long-term survivors with glioma undergo many contrast-enhanced MRI scans, which involve a patient, financial, and environmental burden.

***Findings*** In almost all patients, an increase in T2w/T2w-FLAIR abnormalities was present at the time of tumour progression, mostly but not always accompanying contrast-enhancing findings.

***Clinical relevance*** T2w/T2-FLAIR MRI seems to detect glioma progression in long-term surviving patients similar to contrast-enhanced T1w MRI, raising the question of whether the routine administration of GBCA is necessary and justified in patients under long-term surveillance of glioma.

**Graphical Abstract:**

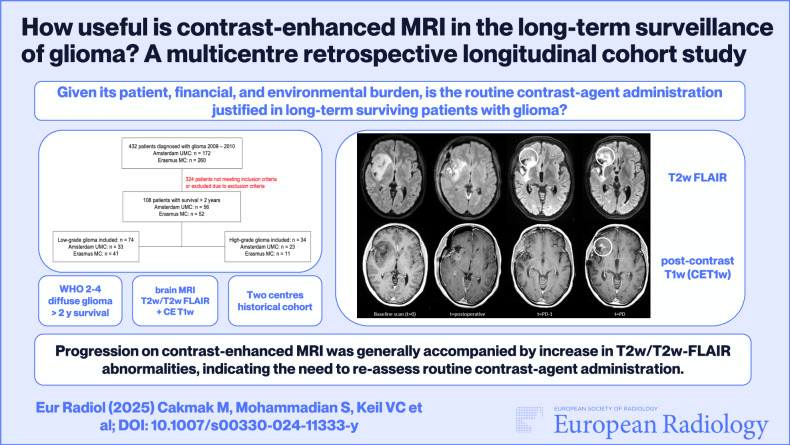

## Introduction

The most prevalent primary brain tumour in adults is adult-type diffuse glioma [1]: about 30% of all brain and central nervous system (CNS) tumours, and 80% of all malignant brain tumours comprise of adult-type diffuse gliomas [2]. The majority of adult-type diffuse glioma are highly malignant World Health Organisation (WHO) grade 4 glioblastoma with a two-year survival rate of 8–12% [3]. On the other hand, low-grade gliomas (LGG) are slow-growing tumours [4]. Routine glioma follow-up consists of magnetic resonance imaging (MRI) containing both (fluid-attenuated inversion recovery (FLAIR)) T2-weighted (T2w) and contrast-enhanced T1-weighted (CET1w) sequences with gadolinium-based contrast agents (GBCA). GBCA are injected intravenously during routine MRI follow-up and help identify progression or dedifferentiation of the tumour based on new or increased enhancement as per the response assessment in neuro-oncology (RANO) criteria [5, 6]. However, from clinical experience, new or increasingly enhancing lesions tend to be accompanied by an increase of abnormalities visible on T2w/T2w-FLAIR MRI [7]. This raises the question of whether the administration of GBCA is always useful or even necessary in the follow-up of patients with glioma. Past concerns with GBCA in terms of nephrotoxicity [8, 9] and neurotoxicity [10] have been addressed by replacing the use of linear with macrocyclic GBCA. Several studies have found deposition of gadolinium in the brain of patients who received GBCA administration, both in glioma tissue and healthy brain tissue, of which the impact is yet unknown [11–13]. The intravenous injection, which is needed to administer GBCA, has been regarded as unpleasant by patients, with anxiety being a prominent adverse event of GBCA administration [14]. Importantly, there is now an increasing awareness of GBCA as a burden on the environment [15].

The burden of GBCA use becomes progressively more evident in patients who are under surveillance for many years and sometimes even decades [16]. Long-term survivors undergo regular follow-up scanning and receive cumulatively large doses of GBCA during the course of their disease [17, 18]. There are currently no evidence-based guidelines on whether or when to stop administering GBCA during prolonged follow-up of patients with glioma. There are no previous studies that investigated whether tumour progression can be determined based on solely T2w abnormalities in these patients, without causing a diagnostic delay.

In this study, we aim to assess whether the routine administration of GBCA in the long-term surveillance of patients with adult-type diffuse glioma is useful. We investigate whether CET1w identifies tumour progression earlier than T2w/T2w-FLAIR MRI only in long-term surviving patients.

## Materials and methods

### Selection of patients

This study was reviewed by the Medical Research Ethics Committees of both medical centres and performed according to the declaration of Helsinki, under the GLIOCARE study protocol in Amsterdam University Medical Centre (AUMC) and the NeuroOnco Biobank in Erasmus MC (EMC). Consent was based on a written informed agreement to allow retrospective data usage. Adult (≥ 18 years of age) patients with pathologically confirmed WHO grade 2–4 adult-type diffuse glioma at AUMC and EMC diagnosed in 2009 or 2010 were retrospectively selected. Patients were included if surgical glioma resection or biopsy had been performed and if they were still alive two years after the surgical procedure. Exclusion criteria were missing or inconclusive radiological or pathological data, refusal to use data retrospectively, retraction or inability to give written informed consent, and the presence of other CNS tumours.

### Data storage and security

Prior to analysis and storage, patient data were coded such that the data could not be directly related to the patient. The key was stored at the imaging trial offices of the Department of Radiology and Nuclear Medicine at AUMC and EMC. Data was entered and stored into an electronic data capture system (Castor EDC https://www.castoredc.com).

### Data extraction

The following clinical data were collected from electronic health records: age, gender, histological tumour grade, tumour molecular status (isocitrate dehydrogenase (IDH) and 1p19q status), type and dates of therapies, and baseline imaging date and characteristics, including the presence of T2w/T2w-FLAIR abnormalities and of enhancement on CET1w MRI. Additionally, follow-up data, including clinical course, survival and radiological findings prior to and upon the time of progression, were collected. This consisted of the identification of an increase of T2w/T2w-FLAIR hyperintensities and new or increasing enhancement on CET1w MRI. Use and total daily dosage of corticosteroids at the time of scanning upon progression, as well as the scan prior to progression, was noted, as corticosteroids can reduce both T2w/T2w-FLAIR abnormalities and enhancement on CET1w MRI thus influencing response assessment. At the time of diagnosis, tumour grade was assessed according to the 2007 WHO classification of tumours of the CNS, since this classification was at that time used in practice [19]. Using the available molecular data, we reclassified the tumours post-hoc to align with the 2021 WHO classification of tumours of the CNS [20]. IDH-wildtype and grade 4 IDH-mutant tumours were categorised as high-grade glioma (HGG), whereas grades 2 and 3 IDH-mutant tumours were categorised as LGG. It should be noted that this classification is based on a combination of molecular markers and histopathological assessment of glioma grade and glioblastoma characteristics.

### Imaging data assessment

Progression after treatment was determined by the treating physician or the multidisciplinary team (MDT) meeting and defined as the moment requiring a change in treatment or follow-up. This included a change in interventions, treatment type or radiological follow-up frequency.

**Fig. 1:**
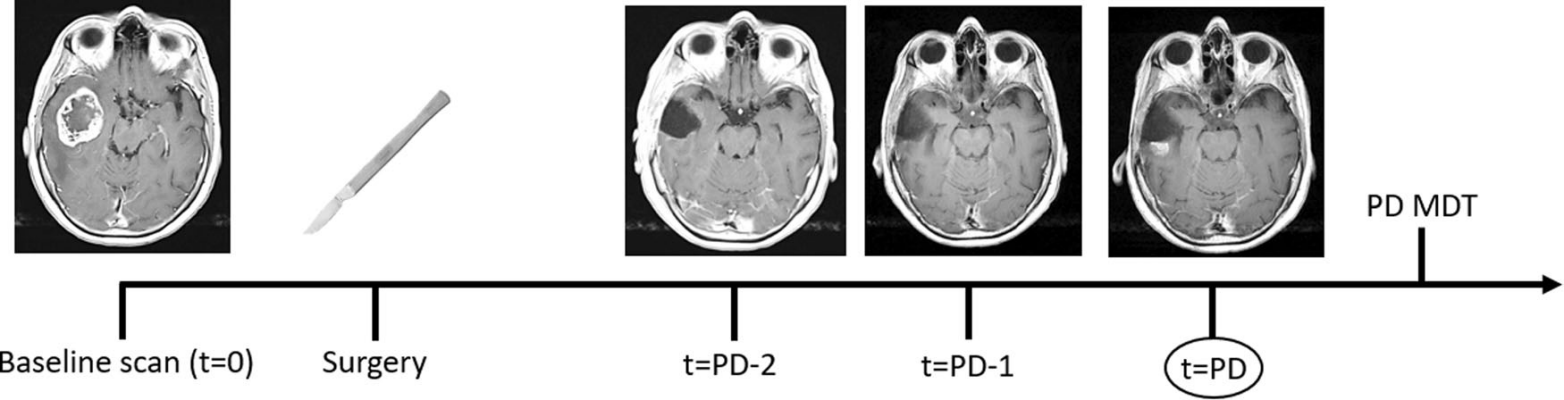
Timeline for a patient illustrating time of progression (PD) as determined in the MDT meeting. One scan prior to the progression scan (at *t* = PD −1) an increase of abnormalities on T2w-FLAIR can already be observed

Baseline imaging was defined as the most recent diagnostic scan performed prior to surgery. This was not necessarily the most recent scan, as (limited) preoperative neuronavigational or functional MRI scans were not considered diagnostic. The baseline scan was used as timepoint zero for calculating time to progression and the total number of scans performed until progression. The MRI scan related to the time of progression was considered the ‘progression scan’ (Fig. 1). From the MRI report, information on tumour size, tumour growth and presence of new lesions was obtained based on T2w/T2w-FLAIR and CET1w MRI. In case this information was unclear or not available in the report, the scans were assessed by an experienced neuroradiologist (V.C.W.K., M.V., and M.S.). With these variables, we determined whether radiological progression was based on the CET1w scan, T2w-/T2w-FLAIR MRI, or both. To determine whether the findings on the progression scan were already present on any earlier scans between the baseline and progression scan, the MRI scans prior to the progression scan were also evaluated. The date of the first mention of an increase in T2w/T2w-FLAIR hyperintensity and/or contrast enhancement was noted. Since both T2w and T2w-FLAIR images are based on the same signal, the first scan, either T2w or T2w-FLAIR, that showed an increase in abnormalities was selected as the first scan with increased abnormalities.

### Analysis

The primary outcome was the proportion of patients that showed an increase of abnormalities on both CET1w and T2w/T2w-FLAIR MRI at the time of progression. Secondary outcomes included the proportion of patients per tumour type/grade that showed progression during their radiological follow-up, the proportion of patients in whom an increase of T2w/T2w-FLAIR abnormalities was already visible on scans prior to the progression scan, the average number of scans during follow-up per tumour type/grade, and the median time to progression per tumour type/grade.

To analyse the relationship between the increase of abnormalities detected on CET1w MRI and the increase of abnormalities detected on T2w/T2w-FLAIR MRI, a chi-square test was performed. A *Phi* coefficient was calculated to determine the degree of association between the two scan types.

## Results

### Patients characteristics

**Fig. 2:**
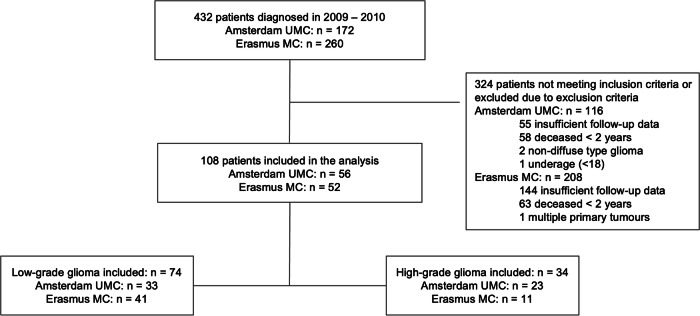
Patient selection

Of the 432 patients who were diagnosed in 2009 and 2010, 308 met the inclusion criteria (Fig. 2). Exclusion criteria were present in 200 patients (primarily lack of sufficient follow-up data available at our centres: *n* = 199); hence 108 patients were considered for analysis: 56 patients from AUMC and 52 from EMC. Patient characteristics are summarised in Table 1. 74 patients (68.5%) had an LGG and 34 patients (31.5%) had an HGG, which were enhanced at baseline in 35 (47.3%) respectively 33 (97.1%) of patients (Table 2a). The cohort included 58 (53.7%) males and 50 (46.3%) females, with a median age at diagnosis of 45 years (range 20–75 years).

**Table 1:** Patient characteristics

Characteristics	
Inclusion per centre	
Amsterdam UMC, *n* (%)	56 (51.9%)
Erasmus MC, *n* (%)	52 (48.1%)
Age at diagnosis	
Mean ± SD (years)	45 ± 14
Range (years)	20–75
Gender	
Female, *n* (%)	50 (46.3%)
Male, *n* (%)	58 (53.7%)
IDH status	
IDH-mutant grade 2/3, *n* (%)	74 (68.5%)
IDH-mutant grade 4, *n* (%)	5 (4.6%)
IDH-wildtype, *n* (%)	29 (26.9%)
Type/grade of tumour (WHO 2021)	
Astrocytoma	
Grade 2, *n* (%)	27 (25%)
Grade 3, *n* (%)	11 (10.2%)
Grade 4, *n* (%)	5 (4.6%)
Oligodendroglioma	
Grade 2, *n* (%)	26 (24.1%)
Grade 3, *n* (%)	10 (9.3%)
Glioblastoma	
Grade 4, *n* (%)	29 (26.9%)

**Table 2:** Absence (−) or presence (+) of enhancement in low (LGG) and high (HGG) grade glioma at baseline (a) and upon progression (b)

	LGG (*n*)	HGG (*n*)
(**a**)		
Baseline scan enhancement −	39	1
Baseline scan enhancement +	35	33
(**b**)		
Baseline scan enhancement − Progression scan enhancement −	13	1
Baseline scan enhancement − Progression scan enhancement +	12	0
Baseline scan enhancement + Progression scan enhancement −	6	0
Baseline scan enhancement + Progression scan enhancement +	22	28

### Imaging abnormalities at the time of progression

**Fig. 3:**
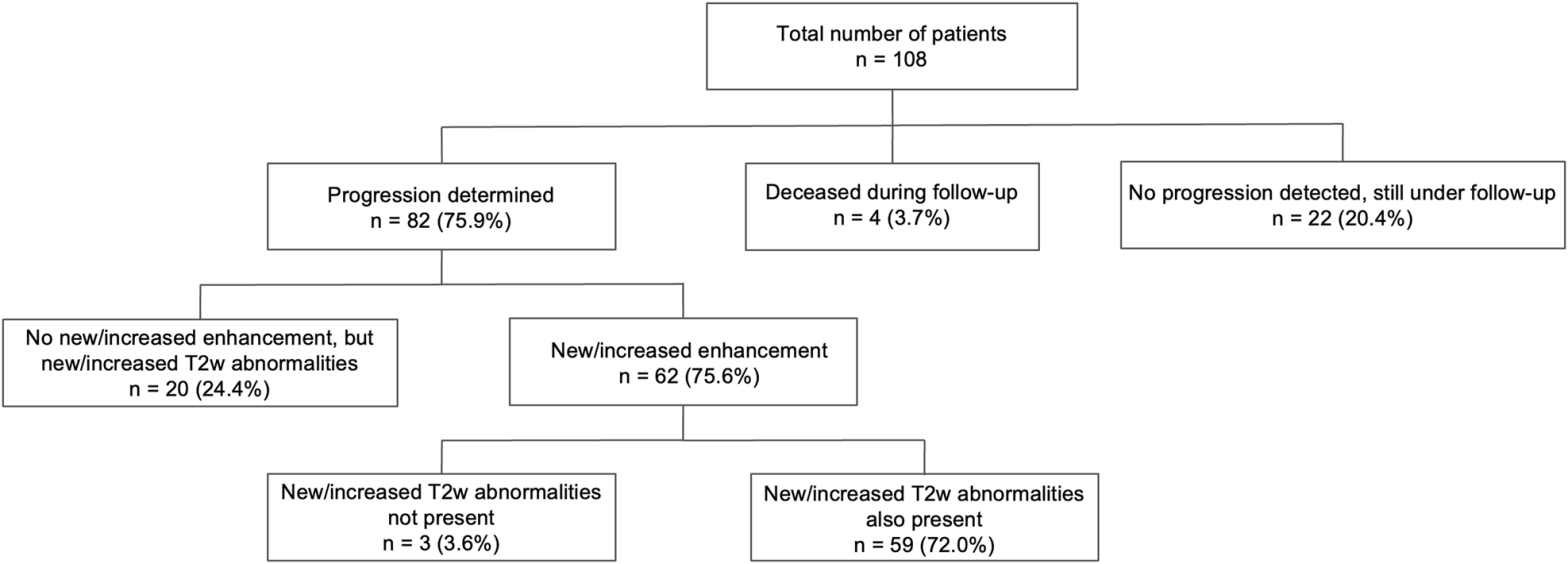
Numbers of patients with progression

Of the 108 included patients, progression during follow-up was determined in 82 patients (75.9%); 4 patients (3.7%) passed away during their follow-up without radiological progression and 22 patients (20.4%) are still under radiological follow-up since their initial diagnosis. Of the 82 patients that showed progression, 59 patients (72.0%) showed both new/increased enhancement on CET1w MRI and new/increased hyperintensities on T2w/T2w-FLAIR MRI. Furthermore, in 20 patients (24.4%) progression was determined solely based on an increase in T2w abnormalities without new nor increased contrast enhancement. Conversely, in three patients (3.6%), progression was determined solely based on CET1w MRI findings. Thus, in 79 out of 82 cases (96.3%), progression could be detected based on increased T2w/T2w-FLAIR abnormalities alone (Fig. 3).

Focusing only on the patients diagnosed with LGG, 53 out of 74 patients showed radiological progression during their follow-up (71.6%) (Table 2a), whereas 21 out of 74 patients are still under follow-up (28.4%). Out of these 53 patients, 31 (58.5%) showed both new/increased enhancement on the CET1w images and new/increased abnormalities on the T2w/T2w-FLAIR images. In 19 patients (35.8%), progression was determined solely on the basis of T2w abnormalities without new nor increased contrast enhancement. Finally, in three patients (5.7%), progression was determined solely based on CET1w. Thus, in 50 out of 53 patients with LGG (94.3%), progression could be detected based on increased T2w/T2w-FLAIR abnormalities alone.

**Fig. 4:**
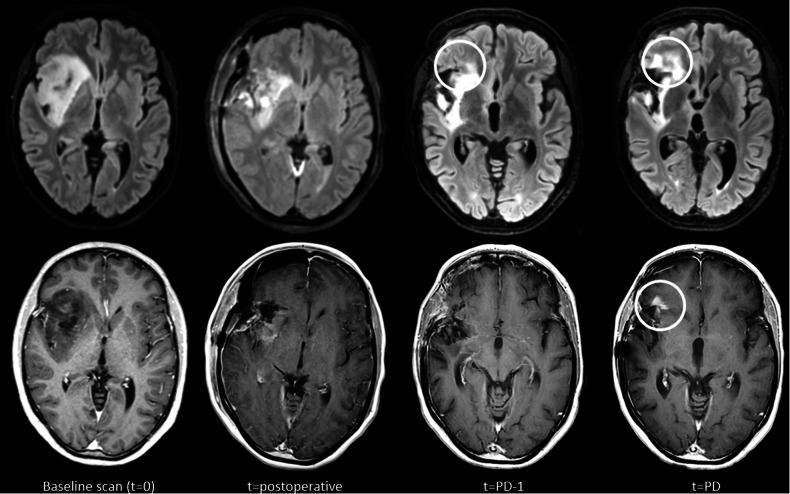
Radiological follow-up of an exemplary patient, showing increased enhancement on the CET1w image and increased hyperintensity on the T2w-FLAIR image at the time of progression (PD), as well as earlier increased abnormalities on the T2w-FLAIR image preceding new contrast enhancement

Finally, in 36 (26 with LGG, 10 with HGG) patients (43.9%) with progression an increase in abnormalities on the T2w/T2w-FLAIR was already seen on scans at a median of 241 days (228 days in LGG, 373 days in HGG) prior to the progression scan (see Fig. 4 for an exemplary patient). Seventeen (13 with LGG, 4 with HGG) patients showed increased abnormalities 1 scan before the progression scan, 4 (3 with LGG, 1 with HGG) patients showed increased abnormalities 2 scans before the progression scan, and 15 (10 with LGG, 5 with HGG) patients showed increased abnormalities 3 or more scans before the progression scan.

Five patients (4.6%) used corticosteroids at the time of the scan prior to the progression scan and one additional patient used corticosteroids (8 mg) at the time of the progression scan but not before. In two patients, the daily dosage was the same at the time of the scan prior to the progression scan and the progression scan (*n* = 1, 0.5 mg; *n* = 1, 4.0 mg). In one patient the daily dosage was higher at the time of progression (6.0 mg) than at the time of the scan prior to the progression scan (4.0 mg). In two patients (1.9%), the daily dosage was lower at the time of progression (*n* = 1, 0.0 mg; *n* = 1, 2.0 mg) than at the time of the scan prior to the progression scan (both 4.0 mg).

### Median time to progression and number of follow-up scans

**Fig. 5:**
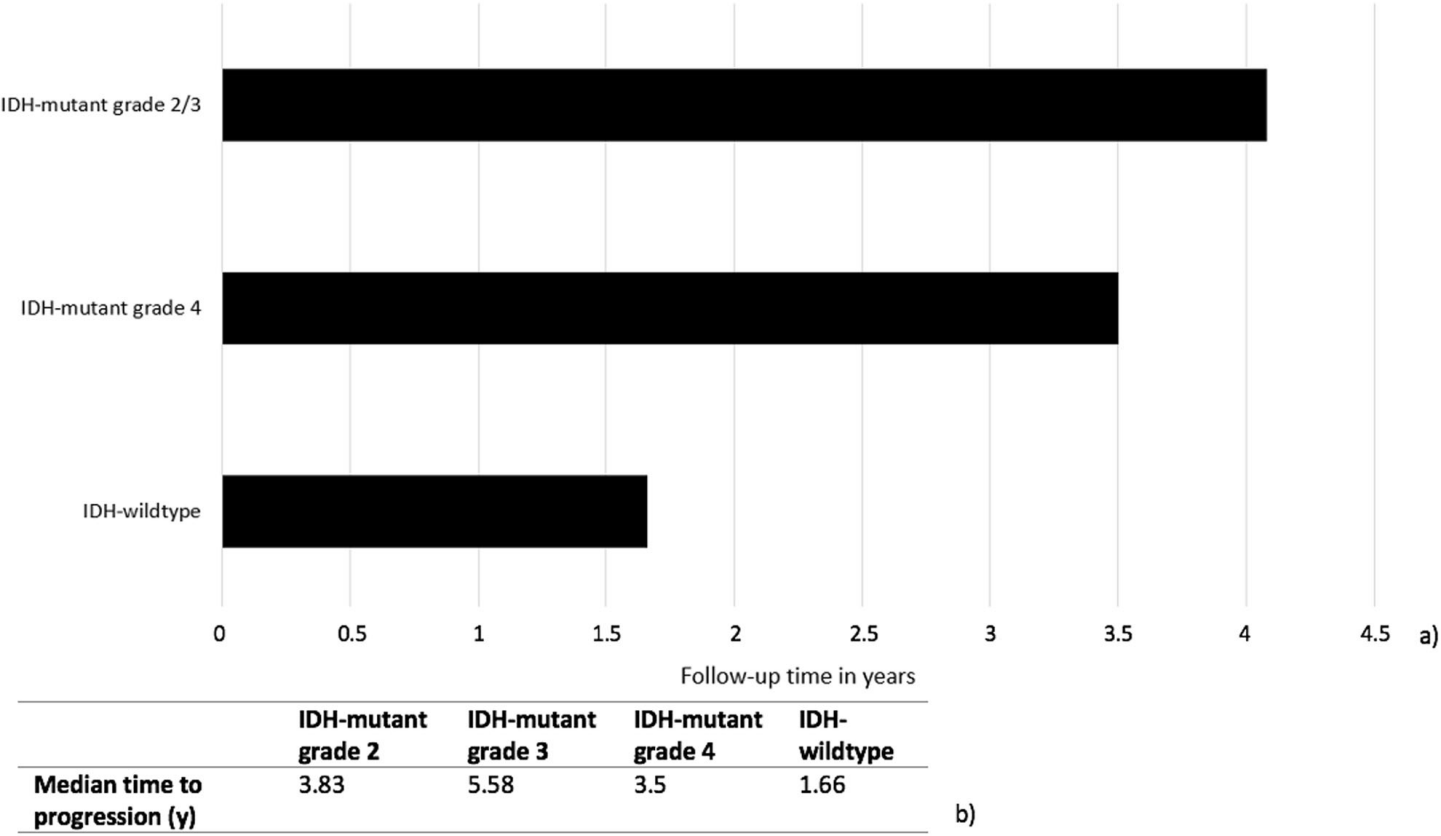
Median time to progression per tumour type (**a**) as detailed per grade in **b**

The median follow-up time until progression per tumour type/grade is shown in Fig. 5. For grade 2/3 IDH-mutant glioma, the median time to progression amounted to 4 years and 1 month (range 9 months to 12 years and 9 months). For grade 4 IDH-mutant glioma, the median time to progression was 3 years and 6 months (range 1 year and 3 months to 5 years and 2 months). Finally, for grade 4 IDH-wildtype glioma, the median time to progression was 1 year and 8 months (range 2 months to 5 years and 2 months).

The number of follow-up MRI scans until progression ranged between 2 and 36 scans. The mean number of scans until progression for grade 2/3 IDH-mutant glioma was 10 (range 2–36), for grade 4 IDH-mutant glioma 14 (range 8–24), and for grade 4 IDH-wildtype glioma 7 (range 2–15). In general, patients with LGG had a follow-up frequency of one scan every 6 months. Patients with HGG had a follow-up frequency of one scan every 3 months.

### Statistical analysis

A chi-square test between an increase in CET1w abnormalities and the T2w/T2w-FLAIR abnormalities revealed a significant *p*-value (*X*^*2*^ (1, *N* = 108) = 23.102, *p* < 0.001). The *Phi*-coefficient was 0.486.

## Discussion

With this study, we aimed to determine the usefulness of contrast-enhanced MRI in the long-term surveillance of patients with glioma for the detection of progression. We found that in almost all cases an increase in CET1w abnormalities was accompanied by an increase in T2w/T2w-FLAIR abnormalities, which raises the question of whether the routine administration of GBCA is always necessary in long-term survivors of glioma.

Progression could be detected in 96.3% of cases based on T2w/T2w-FLAIR imaging abnormalities, compared to 75.6% with CET1w MRI. There was a strong and significant correlation between CET1w and T2w/T2w-FLAIR MRI findings. Twenty patients, 19 of whom with LGG, were diagnosed with progression based on solely T2w/T2w-FLAIR abnormalities, without new or increased enhancement, while only three patients were diagnosed with progression based on CET1w findings only. These data are particularly relevant for patients with LGG, who were shown to have a substantially longer follow-up time until progression compared to patients with HGG, thus receiving many GBCA administrations during their radiological follow-up. Here, we specifically focused on patients with glioma who were still alive two years after diagnosis. This cut-off value is fairly arbitrary but was chosen to be beyond the median survival of glioblastoma. Furthermore, it is in these first two years that tumour behaviour is first assessed and response to treatment is closely monitored. Once patients have remained stable for a longer period, the question of whether repeat GBCA administration is needed becomes particularly relevant.

There is some previous literature on the use of GBCA in neuro-oncology, however, none specifically looked at this patient population. Several studies looked at methods to either reduce or fully eliminate the usage of GBCA. Ammari et al used T2w-FLAIR imaging and CET1w imaging with a 25% dosage of GBCA and created a deep-learning model to create synthetic postcontrast images [21]. These predicted virtual contrast-enhanced images showed a similarity index of 87.1% compared to the native test images, indicating that T2w-FLAIR images can be used to reduce GBCA usage, while still creating high-quality images. Wang et al went a step further and completely eliminated GBCA in their deep learning model, using only T2w-FLAIR images to successfully synthesise virtual contrast-enhanced images [22]. These studies show the potential of using T2w/T2-FLAIR images instead of CET1w images, reducing the usage of GBCA. However, they only looked at replicating CET1w images and the ability to identify existing structures. Furthermore, both studies included different types of malignancies, including meningioma and brain metastasis.

In almost half of the cases (44%) that showed progression, increased abnormalities were already observed on T2w/T2w-FLAIR MRI before progression was determined. These first increases in abnormalities were observed at a median of about 8 months before progression was determined. A previous study that investigated the earliest radiological progression in patients with glioblastoma to determine progression resulted in similar findings [23]. A somewhat counterintuitive finding was, that this time interval was longer in patients with HGG than those with LGG. It should be noted that this was a selected group of patients, while the majority of patients with HGG in fact had increased T2w/T2w-FLAIR abnormalities at the time of progression as expected. This finding can therefore certainly not be generalised to all patients with HGG. Also, this doesn’t mean that an increase in abnormalities on the T2w/T2w-FLAIR MRI can diagnose progression earlier or more accurately. Treatment-related effects without tumour progression, such as post-radiation gliosis, could for instance also underlie an increase in T2 hyperintensity. This finding could however be considered for surveillance strategies without routine GBCA administration as an early marker of change and thus as an indication to add CET1w upon follow-up imaging. Additionally, it should be noted that leptomeningeal seeding could be missed without GBCA administration.

This study was a multicentre longitudinal cohort study, which allowed us to better understand the radiological follow-up of patients with glioma over time. The retrospective design prevented bias from loss of follow-up. Since the patients that were selected were diagnosed in 2009 or 2010, we were able to track the extensive amount of radiological follow-up per patient, with some patients still under surveillance as of 2024, 14–15 years after their initial diagnosis. Some patients only developed progression after many years of follow-up, having had GBCA administered during every single scan, providing insight into the burden on both the patient and the environment of routine GBCA use.

This study met with a few limitations. This study was based on data collection from medical records of patients that were maintained under follow-up at our institutions and therefore susceptible to selection bias. Furthermore, the conclusions were drawn based on radiological reports, which are based on the expertise of the radiologist. However, this is the real-life setting in which the patients were also diagnosed at the time. A small number of patients used corticosteroids at the time of progression, which could have influenced the extent of the imaging findings, particularly in those cases when the dosage was lower at the time of progression: a reduction of the influence of steroids on T2-abnormalities or enhancement could thus have potentially led to a false interpretation of progression of findings on MRI. This constituted only 1.9% of the included patients with thus only a very limited impact on the overall findings. Finally, the retrospective nature of the study meant that imaging sequences and timing of imaging were not standardised. This heterogeneity results in some uncertainty regarding the exact timing of tumour progression across patients.

Our work provides the first indication that GBCA administration may not be routinely useful in long-term survivors of glioma. However, additional research is needed to determine whether and if so when to stop or reduce (through intermittent) GBCA administration in long-term survivors of glioma. Treatment decisions will likely continue to require confirmation of tumour progression with GBCA-enhanced imaging. A larger sample size is also recommended such that subgroup analyses can be performed, for instance, based on tumour grade, (molecular) type or MRI characteristics (e.g., presence or absence of contrast-enhancement). Moreover, additional non-contrast enhanced sequences other than T2w/T2w-FLAIR could be considered that may aid in the diagnostic accuracy of tumour progression. Perfusion-weighted imaging with arterial spin labelling, for instance, provides additional parameters that can help with the identification of tumour progression and has been increasingly utilised in the everyday practice of neuro-radiologists [24]. More novel techniques, such as relaxometry [25] or chemical exchange saturation transfer (CEST) [26] MRI, may also provide information on tissue characteristics that may indicate tumour activity without GBCA administration. Additionally, virtual contrast-enhanced images provided by deep learning algorithms as described above could be incorporated in future, prospective observational or even randomised controlled studies.

## Conclusion

In conclusion, our study provides important insight into MRI progression patterns in patients surviving long-term with glioma. We found that T2w/T2-FLAIR abnormalities seem to detect progression in these patients similar to CET1w MRI, raising the question of whether current surveillance strategies, particularly for patients with long-term follow-up, with routine administration should be re-assessed.

## Abbreviations

AUMC: Amsterdam University Medical Centre
CET1w: Contrast-enhanced T1-weighted
CNS: Central nervous system
EMC: Erasmus Medical Centre
FLAIR: Fluid-attenuated inversion recovery
GBCA: Gadolinium-based contrast agents
HGG: High-grade glioma
IDH: Isocitrate dehydrogenase
LGG: Low-grade glioma
MDT: Multidisciplinary team
MRI: Magnetic resonance imaging
T2w: T2-weighted
WHO: World Health Organisation
